# Translation and validation of non-English versions of the Ankylosing Spondylitis Quality of Life (ASQOL) questionnaire

**DOI:** 10.1186/1477-7525-5-7

**Published:** 2007-02-02

**Authors:** Lynda C Doward, Stephen P McKenna, David M Meads, James Twiss, Dennis Revicki, Robert L Wong, Michelle P Luo

**Affiliations:** 1 Galen Research, Manchester UK; 2 Center for Health Outcomes Research, United Biosource Corporation, Bethesda, MD, USA; 3 Abbott Laboratories, Parsippany, NJ, USA; 4 Abbott Laboratories, Abbott Park, IL, USA

## Abstract

**Background:**

The Ankylosing Spondylitis Quality of Life (ASQOL) questionnaire is a unidimensional, disease-specific measure developed in the UK and the Netherlands. This study describes its adaptation into other languages.

**Methods:**

The UK English ASQOL was translated into US English; Canadian French and English; French; German; Italian; Spanish; and Swedish (dual-panel methods). Cognitive debriefing interviews were conducted with AS patients. Psychometric/scaling properties were assessed using data from two Phase III studies of adalimumab. Baseline and Week-2 data were used to assess test-retest reliability. Validity was determined by correlation of ASQOL with SF-36 and BASFI and by discriminative ability of ASQOL based on disease severity. Item response theory (Rasch model) was used to test ASQOL's scaling properties.

**Results:**

Cognitive debriefing showed the new ASQOL versions to be clear, relevant and comprehensive. Sample sizes varied, but were sufficient for: psychometric/scaling assessment for US English and Canadian English; psychometric but not scaling analyses for German; and preliminary evidence of these properties for the remaining languages. Test-retest reliability and Cronbach's alpha coefficients were high: US English (0.85, 0.85), Canadian English (0.87, 0.86), and German (0.77, 0.79). Correlations of ASQOL with SF-36 and BASFI for US English, Canadian English, and German measures were moderate, but ASQOL discriminated between patients based on perceived disease severities (p < 0.01). Results were comparable for the other languages. US English and Canadian English exhibited fit to the Rasch model (non-significant p-values: 0.54, 0.68), confirming unidimensionality.

**Conclusion:**

The ASQOL was successfully translated into all eight languages. Psychometric properties were excellent for US English, Canadian English, and German, and extremely promising for the other languages.

## Background

Ankylosing spondylitis (AS) is a chronic rheumatic disease with a worldwide prevalence of up to 0.9% [1] and rates as high as 2.0% reported for some ethnic groups [2]. The sacroiliac joints are affected and, to a varying degree, the spinal column. The disease may also involve the peripheral joints and extra-articular structures [1]. Patients commonly experience pain, morning stiffness and disability, all which generally increase with duration of disease [3]. Systemic features, such as anorexia and fatigue may also occur. In late disease, some patients develop acute anterior uveitis, cardiovascular or pulmonary problems [2]. Men are more commonly affected than women [4] and, as with other sponylarthritides, AS is associated with positivity for the HLA-B27 gene [1].

AS typically emerges during the second or third decade of life, with the peak age of onset in the mid-20s [4]. The condition impacts many areas of patients' lives, with symptomatic problems affecting mood, motivation and ability to cope with day-to-day activities [5]. Social and work-related problems have been reported along with adverse effects on patients' abilities to meet their needs for stimulation, exploration and role fulfilment [1,5,6]. Reduced self-image, self-esteem and feelings of worth have also been reported [5]. The collective impact of AS has a substantial influence on patients' quality of life (QOL). As the condition emerges in early adulthood, its effects are present for much of patients' lives.

The Ankylosing Spondylitis Quality of Life (ASQOL) questionnaire is an AS-specific measure of QOL [5]. The instrument was designed for monitoring patients and evaluating treatment effects of new pharmaceutical products from the patients' perspectives. It adopts the needs-based model of QOL that postulates that life gains its quality from the ability of individuals to satisfy their own needs [7]. The model is well-established and has been applied successfully in the development of a large number of disease-specific QOL instruments, several of which have become established as the outcome instrument of choice for clinical trials and studies [8-17].

The development methodology was based on advances in the understanding of the conceptual and practical basis of measurement; combining the theoretical strengths of the needs-based QOL model with the statistical and diagnostic power of the Rasch model [18]. The application of the Rasch model ensures that the fundamental scaling properties of the instrument were assessed in addition to the traditional psychometric properties. Content was derived directly from interviews with AS patients, and extensive assessments have confirmed that the instrument has excellent psychometric and scaling properties [5]. The ASQOL has been previously reviewed by the Assessments in AS International Working Group studies. It is the most thoroughly studied of the AS-specific QOL instruments available and has been successfully employed in a number of clinical trials [19-21]. The instrument was originally developed in parallel in the United Kingdom and the Netherlands and has subsequently been adopted for use in Hungary [22]. The purpose of the current study was to produce validated and reliable new language versions for the United States (English), Canada (French and English), France, Germany, Italy, Spain and Sweden for use in clinical trials with the tumour necrosis factor antagonist adalimumab [23,24].

## Methods

### Patients

Patients from the United States, Canada, Germany, France, Italy, Spain, and Sweden were eligible for study participation if they were adults (>18 years age) with a confirmed diagnosis of AS. Potential participants were excluded if they reported a co-morbidity or displayed any cognitive, psychological or physical impairment that, in the investigators' opinions, would interfere with their participation in the study. Patients were recruited via local participating clinical centres. Patients participating in cognitive debriefing were also recruited via advertisements or patient support groups. All centres received approval from independent ethics committees, and the study was conducted in accordance with the ethics principles of the Declaration of Helsinki. Investigators assured that the study complied with prevailing local laws and customs. Written informed consent was obtained from all respondents prior to the start of their participation.

### Language translation

The ASQOL was translated using the dual-panel approach [25]. This method has been uniformly employed in the development of new language versions of needs-based questionnaires including the ASQOL and assumes that the verification and acceptability of translations should rest with people typical of future respondents [25,26]. Translation involves running two panels (a bilingual and a lay translation panel) in each target country. Working as a group, the bilingual panel produce an initial translation for consideration by the lay panel. The lay panel is composed of individuals of average or lower educational levels, who consider the draft translation to ensure the content is expressed in clear, everyday language. When the instrument is required for use in another English-speaking population, only the lay panel is required.

### Cognitive debriefing interviews

Cognitive debriefing interviews were conducted with AS patients in each country to test the acceptability, comprehensibility, relevance and completeness of the new translations. Participants completed the ASQOL in the presence of an interviewer and were then invited to comment on the items, instructions and response format.

### Assessment of psychometric and scaling properties

Data used to evaluate the psychometric properties of the new ASQOL language versions were collected from two 24-week, randomised, placebo-controlled, double-blind, Phase III studies involving adalimumab conducted in the United States and Europe (Study M03-607 known as ATLAS [23]) and in Canada (Study M03-606 [24]). ASQOL data collected at baseline, 2 weeks, and 12 weeks after randomisation were analyzed.

### Study instruments

The ASQOL comprises 18 questions, each with a dichotomous "yes/no" response format, scored "1" and "0," respectively. Total scores range from 0–18, with a higher score indicating poor quality of life. The Bath Ankylosing Spondylitis Functional Index (BASFI) [27] assesses functional limitations in AS via 10 questions, each employing a 10-cm visual analogue scale (VAS). A mean of the scores for the 10 items provides the total BASFI score, with values ranging from 0–10. A high BASFI score indicates greater functional limitation. The Bath Ankylosing Spondylitis Disease Activity Index (BASDAI) [28] uses six 10-cm VAS scales to determine the severity of five major symptoms of AS. A mean of the scores for the six items provides the total BASDAI score. Again, scores range from 0–10, with a higher score indicating greater disability. The Short Form 36 Health Survey (SF-36) [29] is a general health-status questionnaire comprising 36 items in eight domains of physical and mental health: physical functioning, social functioning, role-physical, role-emotional, mental health, vitality, pain and general health perception. Physical and Mental Component Summary scores (PCS and MCS) are calculated by summing domain items and transforming summations onto a scale from "0" or "worst health" to "100 or "best health." Patient's Global Assessment of Disease Activity VAS (termed "patient VAS" below) and a Physician's Global Assessment of Disease Activity VAS (termed "physician VAS" below) took the form of a horizontal line marked from 0 to 100. A higher score indicated greater rated severity.

### Assessment of traditional psychometric properties

Test-retest reliability (reproducibility) was assessed by correlating baseline and Week-2 ASQOL scores using Spearman rank correlation coefficients. Reproducibility is best evaluated by focusing on patients with relatively stable disease conditions over time. Therefore, patients were excluded from these analyses if they reported significant changes (≥ 30%) in patient or physician VAS scores. Values greater than 0.85 for rank correlation coefficients were considered evidence of high reliability [30,31].

Internal consistency was assessed using Cronbach's alpha coefficients, for which values greater than 0.70 were considered evidence of internal consistency [31]. Construct validity was tested by assessing convergent and discriminative validity. For convergent validity, association between ASQOL and the SF-36 domain scales, the BASFI and the patient and physician VAS responses was assessed by Spearman rank correlation coefficients. Moderate correlations were expected, indicating that the scales assess different but related constructs. Discriminative validity was assessed by testing the ability of the ASQOL to discriminate between groups based on severity of illness (physician and patient VAS scores), disease activity (BASDAI) and general health status (i.e., SF-36 Question 1: In general would you say your health is; Excellent, Very good, Good, Fair, or Poor?).

### Assessment of the scaling properties of the ASQOL

Rasch analysis was conducted to assess scale unidimensionality (i.e., that items assess a single underlying construct) and item hierarchical ordering (i.e., items represent different amounts of the construct assessed). In addition, differential item functioning or DIF was assessed to ensure items worked in the same manner across different patient groups and to ensure item stability, to provide additional evidence of scale reliability. The model used was the one-parameter logistic item response theory (IRT) model [18].

Analyses were conducted using the Rasch Unidimensional Measurement Model (RUMM2020) software [32]. This uses the pairwise maximum likelihood estimation procedure. If data from the scale are shown to fit the model, the property of unidimensionality is confirmed. The adequacy of the fit of the ASQOL to the model was evaluated through a total Chi-square (χ^2^) fit statistic. A non-statistically significant χ^2 ^is taken as evidence of fit to the model. The logit and person coverage of the ASQOL was examined using the item-location and person-location distribution maps. This provides information on item ordering, level of QOL assessed by individual items, and the scope of the underlying construct covered by the scales. DIF was assessed across age, gender and disease duration (above or below the median) subgroups using analysis of variance (ANOVA) models. The stability of the items over time was evaluated by examining each item for DIF by administration. Items that exhibited DIF by administration cannot be considered stable over time, which would call into question the scale's reproducibility. In addition, the stability of the item logit locations was assessed over time. Items whose logit location values were more than two standard errors (SE) apart at Time 1 and Time 2 would be considered statistically significantly different, and, therefore, item stability would not be considered present [33].

### Sample size

Which of the specific analyses described above were undertaken for each ASQOL language version were determined by the country sample size. Traditional statistical analyses generally employ sample sizes of 40 or more. For Rasch analysis, data from 50 respondents allows 99% confidence that the item calibrations are stable within ± 1 logit [34].

## Results

### Translation

The translation panels (both bilingual and lay) consisted of four to six participants each, with approximately equal numbers of men and women. Most items in the questionnaire were capable of translation into all eight languages without difficulty. As the emphasis was on conceptual rather than linguistic translations, some of the translated items took a different form from the UK original. The US and Canadian English panels replaced colloquial UK expressions with ones more suitable for the source culture throughout the questionnaire.

### Cognitive debriefing interviews

Interviews were conducted with 12–16 patients in each country. The samples contained broad ranges of age (18–82 years) and disease duration (1–52 years). The samples were predominantly men in all countries except Spain, where equal numbers of men and women were interviewed. Based on their responses, participants found the new language versions to be clear, unambiguous, comprehensive and easy to complete. Completion times ranged from 1.5–12 minutes (mean 5.8 minutes). All language versions were considered relevant to patients with AS in the target countries. No changes were required to any of the individual language versions as a result of the debriefing exercise.

### Assessment of psychometric and scaling properties

Participant details are shown in Table 1. Sample size varied considerably by country. Those in the USA and Canada (English language version) were sufficient to permit assessment of psychometric and scaling properties. The Germany sample was sufficient to allow traditional psychometric analyses but not assessment of scaling properties. Samples for the remaining countries were relatively small and are therefore presented as preliminary evidence of these properties. Further studies will be required to confirm the properties of these language versions.

**Table 1:** Sample Details for Psychometric and Scaling Data

		**US (N = 148)**	**Canadian English** **(N = 66)**	**Canadian French** **(N = 16)**	**French** **(N = 18)**	**German** **(N = 37)**	**Italian** **(N = 22)**	**Spanish** **(n = 24)**	**Swedish** **(n = 9)**
**Gender**	**Male (%)**	111 (75.0)	51 (77.3)	14 (87.5)	12 (66.7)	26 (70.3)	16 (72.7)	19 (79.2)	8 (88.9)
	**Female (%)**	37 (25.0)	15 (22.7)	2 (12.5)	6 (33.3)	11 (29.7)	6 (27.3)	5 (20.8)	1 (11.1)
**Age**	**Mean (SD)**	44.7 (12.5)	39.82(10.5)	45.3 (12.0)	35.7 (8.0)	41.9 (11.8)	40.7 (10.8)	38.0 (9.0)	37.6 (9.1)
	**Median (IQR)**	46.0 (34.0–55.0)	41.0 (32.0–45.0)	44.5 (38.3–53.8)	34.5 (30.3–42.0)	42.0 (33.0–52.5)	44.0 (34.5–49.0)	8.6 (5.7 – 17.1)	36.0 (30.5 – 45.5)
	**Range**	18.0–71.0	18.0–69.0	28.0–69.0	25.0–55.0	23.0–62.0	21.0–58.0	3.3 – 37.4	24.0 – 52.0
**Disease duration**	**Mean (SD)**	11.0 (10.3)	12.3 (7.5)	17.0 (7.5)	9.3 (8.0)	9.7 (9.1)	9.2 (9.4)	12.2 (8.9)	11.4 (9.4)
	**Median (IQR)**	7.6 (2.4–18.3)	11.6 (5.4–18.1)	16.3 (10.3–22.6)	9.8 (4.3–13.7)	6.2 (1.9–15.4)	4.9 (1.2–18.3)	8.6 (5.7 – 17.1)	11.7 (0.5 – 19.8)
	**Range**	0.1–48.4	0.2–40.3	5.2–30.1	0.2–17.4	0.2–35.3	0.1–28.4	3.3 – 37.4	0.1 – 25.3

IQR = interquartile Range: the distance between the 75th percentile and the 25th percentile.

Table 2 shows scores on selected instruments at baseline. The samples covered broad ranges of disease duration and severity. A majority of patients viewed themselves as having "good" or "fair" health as rated by the SF-36 general health question. There were some differences observed between physician and patient VAS scales, with the greatest disparity evident between the United States and Sweden (possibly a result of the small sample size in the latter country). Ceiling effects for the ASQOL in all countries were minimal, with the highest effect in the United States (4.7% obtained the maximum score).

**Table 2:** Baseline Instrument Scores by Country

		**US (N = 148)**	**Canadian English** **(N = 66)**	**Canadian French** **(N = 16)**	**French** **(N = 18)**	**German** **(N = 37)**	**Italian** **(N = 22)**	**Spanish** **(N = 24)**	**Swedish** **(N = 9)**
ASQOL	Mean (SD)	9.5 (4.4)	11.4 (4.4)	10.9 (3.6)	10.5 (3.9)	10.6 (3.7)	12.1 (4.5)	11.8 (4.1)	7.2 (3.9)
	Median (IQR)	10.0 (6.0–10.0)	12.5 (8.0–15.0)	10.0 (9.3–12.8)	11.5 (9.0–13.0)	11.0 (9.0–14.0)	12.5 (8.8–16.0)	12.5 (9.0–15.0)	7.0 (3.0–10.0)
	Range	1–18	0–18	2–17	2–17	2–18	3–17	3–18	3–14
	% Scoring, min	0.0	1.5	0.0	0.0	0.0	0.0	0.0	0.0
	% Scoring, max	4.7	1.5	0.0	0.0	2.7	0.0	4.2	0.0
Patient VAS*	Mean (SD)	62.8 (21.5)	68.8 (17.6)	59.8 (22.6)	51.8 (19.7)	66.0 (18.9)	69.9 (23.3)	64.2 (18.6)	45.7 (22.3)
	Median (IQR)	66.0 (50.3–79.0)	69.5 (58.8–82.3)	58.0 (46.5–82.5)	46.5 (39.0–66.8)	67.0 (54.0–81.5)	78.0 (58.0–88.0)	66.0 (45.0–79.0)	38.0 (26.5–67.5)
	Range	7–100	19–97	9–92	16–88	19–100	9–97	32–100	18–78
SF-36 Question 1^#^	Excellent (%)	7 (4.7)	0 (0)	0 (0)	0 (0)	0 (0)	0 (0)	0 (0)	0 (0)
	Very Good (%)	18 (12.2%)	9 (13.6%)	1 (6.3%)	0 (0)	1 (2.7%)	0 (0)	0 (0)	4 (44.4%)
	Good (%)	73 (49.3%)	22 (33.3%)	3 (68.8%)	7 (38.9%)	5 (13.5%)	1 (4.5%)	3 (12.5%)	2 (22.2%)
	Fair (%)	38 (25.7%)	25 (37.9%)	11 (18.8%)	8 (44.4%)	22 (59.5%)	10 (45.5%)	10 (41.7%)	1 (11.1%)
	Poor (%)	11 (7.4%)	10 (15.2%)	1 (6.3%)	3 (16.7%)	9 (24.3%)	11 (50.0%)	11 (45.8%)	2 (22.2%)

*Patient VAS = Patient's Global Assessment of Disease Activity Visual Analogue Scale. ^#^SF-36 Question 1: "In general, how would you say your health is: Excellent, Very Good, Good, Fair, or Poor?"

### Traditional Psychometric Properties

The results of the test-retest reliability and internal consistency analyses are shown in Table 3. The US and Canadian (English and French), French and Swedish versions were shown to have adequate test-retest reliability. The reliability for the Italian sample was borderline and the German and Spanish measures showed slightly lower reliability than the desired 0.85 level. However, it must be noted that analysis carried out in countries other than the US and Canada is only preliminary due to the sample sizes and further investigation is needed before any firm conclusions can be made. Cronbach's alpha coefficients were within an acceptable range for all new language versions of the ASQOL.

**Table 3:** ASQOL Test-Retest Reliability and Internal Consistency Values by Country

	**Test-retest reliability**		**Internal Consistency:** **Cronbach's Alpha Coefficient**		
	**N**	Correlation*	**N**	**Baseline**	**Week 12**
**US**	102	.85	148	.85	.92
**Canadian English**	51	.86	66	.86	.91
**Canadian French**	12	.87	16	.44	.83
**French**	12	.96	18	.81	.93
**German**	28	.77	37	.79	.81
**Italian**	12	.84	22	.87	.93
**Spanish**	14	.77	24	.84	.93
**Swedish**	7	.85	9	.81	.86

* Spearman rank

**Figure 1:**
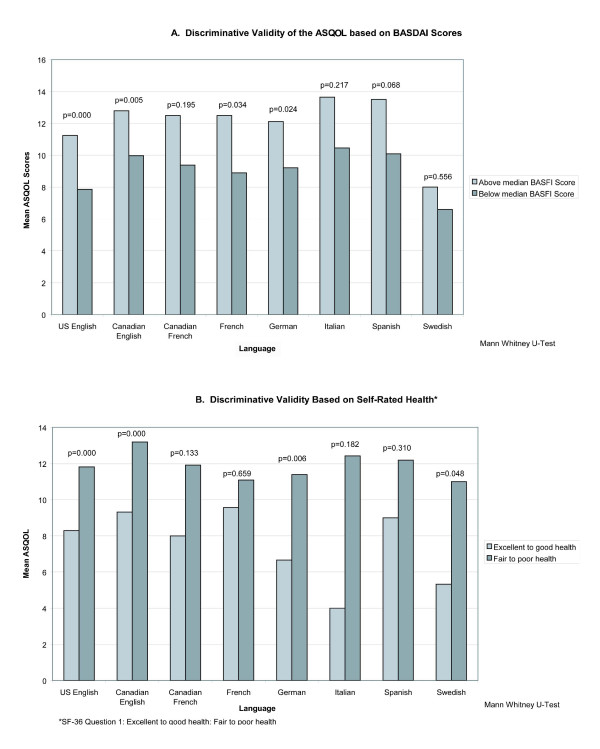
A. Discriminative validity of the ASQOL based on BASDAI scores. B. Discriminative validity based on self-rated health.

Table 4 presents the association between ASQOL and comparator instrument scores at baseline. Observed correlations with the BASFI and SF-36 domains were moderate for the US English, Canadian English and German versions, providing evidence of convergent validity. As the sample sizes for these countries were sufficiently large, confidence can be placed in the results obtained. For the remaining countries, the smaller sample sizes have produced potentially spurious results, and definitive conclusions cannot be drawn. The ASQOL had greater correlations with the patient VAS than physician VAS scores in most countries. Again, this would be expected, as both the patient VAS and the ASQOL were completed by patients and reflected their subjective assessments of their conditions. The ASQOL discriminated between patients' disease activity groups as assessed by the BASDAI. The groups with greater disease activity (above median BASDAI scores) had higher mean ASQOL scores (indicating poorer QOL). These differences were statistically significant for the US, Canadian English, German and French samples (Figure 1A). Figure 1B indicates that the US, Canadian English, German and Swedish versions differentiated between patients on the basis of self-rated health by SF-36 Questions 1.

**Table 4:** Correlations Between ASQOL Scores and Comparator Instruments at Baseline

	**N**	**Correlation of ASQOL and SF-36 Domains**								**Correlation of ASQOL and BASFI**	**Correlation of ASQOL and Physician VAS**	**Correlation of ASQOL and Patient VAS**
		Functioning	Physical Health	Role- Emotional	Energy/Fatigue	Emotional Well-being	Social Functioning	Pain	General Health			
**USA**	148	-.56*	-.59*	-.41*	-.57*	-.49*	-.69*	-.69*	-.50*	.57*	.31*	.61*
**Canadian English**	66	-.70*	-.47*	-.24	-.50*	-.47*	-.67*	-.64*	-.57*	.68*	.31	.36*
**German**	37	-.43*	-.21	-.33	-.54*	-.52*	-.55*	-.63*	-.37	.52	.14	.58*
**Spanish**	24	-.63*	-.40	-.45	-.49	-.62*	-.39	-.39	-.52*	.61*	.43*	.34
**Italian**	22	-.65*	-.31	-.60*	-.27	-.73*	-.56*	-.29	-.61*	.66*	.26	.24
**French**	18	-.31	-.49	-.15	-.57	-.46	-.65*	-.51	-.28	.29	.45	.46
**Canadian French**	16	-.67*	-.44	-.45	-.56	-.36	-.47	-.42	-.26	.64*	.34	.53
**Swedish**	9	-.82*	-.70	.13	-.83*	-.84*	-.54	-.90*	-.55	.74	.31	.49

*Correlation is significant at the 99% level;correlation is significant at the 95% level.

**Figure 2:**
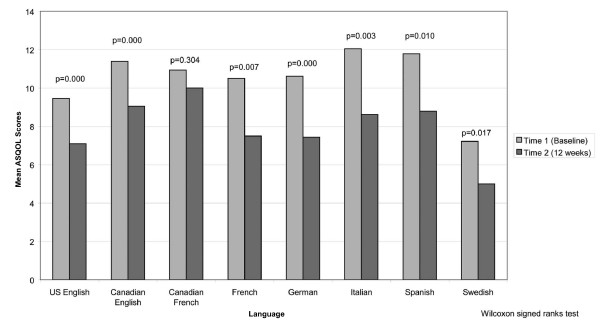
Mean ASQOL scores at baseline (time 1) and 12 weeks (time 2).

Evidence of responsiveness was provided by comparing ASQOL scores at baseline and after 12 weeks of treatment. Figure 2 shows that the mean ASQOL scores are higher at Time 1 than Time 2. This difference was significant for all language versions except the Swedish and Canadian French versions. Again, small sample sizes may explain why significant changes were not observed for these versions. The effect size for this change was large for Germany (0.85) and moderate (0.53–0.77) for the other languages, with the exception of Canadian French (0.26). This suggests that the new ASQOL language versions are responsive to change over time.

### Scaling properties of the ASQOL

Scaling properties were assessed for the US and Canadian English language versions of the ASQOL. Application of Rasch analysis revealed that both versions exhibited sufficient fit to the model (non-significant χ^2 ^p-values: United States, 0.54; Canadian English, 0.68), confirming unidimensionality. The item hierarchy was similar over time for each language version, and logit values were stable over time. The item for both languages representing the greatest degree of the underlying trait measured was Item 16, "I find it difficult to wash my hair." Conversely, the item representing the lowest degree of the underlying trait measured was Item 14, "The pain is always there." Item stability over time was assessed by examining each item for differential item functioning (DIF) based on administration time point. No items in either the US or Canadian English version exhibited significant DIF by administration (p ≤ 0.01), providing evidence of the reliability of both. The person-item location distributions demonstrated that the ASQOL items targeted the respondents relatively well (data not shown).

DIF was also assessed according to demographic factors (age, sex, and duration of disease. For the US data, there was no significant DIF (p < 0.01) according to duration of disease. However, there was uniform DIF observed for one item each related to age and sex, respectively. Women were significantly more likely than men to affirm the item "I sometimes feel like crying." Those who were younger than the median age (46 years) were significantly more likely to affirm the item "I am tired all the time" than those who were older than the median age. No items in the Canadian-English ASQOL exhibited DIF by demographic factors.

## Discussion

The ASQOL was successfully translated into the target languages. The cognitive debriefing interviews suggest that the new language versions have high face and content validity, and are clear, comprehensive and acceptable to patients in each country.

The data available for the assessment of psychometric and scaling properties of the new language versions were generated from a 12-week, multinational clinical trial. As these analyses were retrospective, it was not possible to guarantee that sufficient data were collected in each country. Consequently, small sample sizes were observed in some countries, which precluded the completion of certain analyses for those ASQOL language versions. Test-retest reliability was excellent for those versions with acceptable sample sizes (US and Canadian English).

Preliminary evidence of reliability obtained for the remaining language versions was encouraging. The internal consistency was adequate in all language versions. Correlations between the new ASQOL language versions and the BASFI and SF-36 scales were moderate, indicating that they assess related but distinct concepts. Construct validity was further assessed by examining the ASQOL's ability to distinguish between high-scoring and low-scoring groups on the comparator measures. The Canadian English, US and German ASQOL versions were able to distinguish between groups, providing strong evidence of their discriminative abilities. The French language version showed promising discriminative ability while the Canadian French, Swedish, Italian and Spanish language versions showed limited ability to discriminate between groups. The lack of findings in the latter countries may be explained by the small sample sizes available rather than a shortcoming of the ASQOL language versions.

Further data collection and analyses are required to confirm the discriminative ability of the Canadian French, Swedish, Italian, and Spanish ASQOL language versions. Responsiveness to change was assessed via analysis of change in scores over the 12-week trial period using effect sizes and within-group statistical analyses. Analyses indicated that all new versions of the ASQOL (except Canadian French and Swedish) were responsive to change.

The sample sizes available for the US and Canadian English versions were sufficient to allow assessment of scaling properties. Rasch analyses were performed to examine the item stability of the questionnaires. Analyses indicated that the hierarchy of item ordering was relatively consistent over time for both these versions. In addition, the item logit locations appeared to be relatively stable over time. Examination of DIF showed that these language versions of the ASQOL did not exhibit DIF by administration. The combination of evidence from the Rasch analyses suggests that the Canadian English and US versions of the ASQOL have the property of item stability and are reliable assessments of QOL over time.

The psychometric evaluation of the new language versions of the ASQOL has been largely successful. Based on the research presented here, strong claims can be made for the reliability, validity and responsiveness of the Canadian English and US language versions. The German and French language versions exhibited encouraging validity and responsiveness. Further evidence is needed of the reliability and validity of the Canadian French, Swedish, Italian, and Spanish language versions because of the relatively small sample sizes from those countries.

The authors recommend that ASQOL data continue to be collected for all language versions, to provide further evidence of their psychometric properties – particularly for the Canadian French, French, Swedish, Italian, and Spanish language versions.

## Competing interests

Authors Doward and McKenna led the team that developed the ASQOL measure, under funding from the UK National Health Service Research & Development Executive. Galen Research (authors Doward, McKenna, Meads, and Twiss) received financial support for the current study from Abbott Laboratories. Author Revicki received research funding support from Abbott as well. He declares no other competing interests. Authors Wong and Luo are Abbott employees.

## Authors' contributions

LCD designed and coordinated the study, managed translation and cognitive debriefing activities for all European countries, participated in statistical analysis, and drafted the manuscript. SPM co-designed and coordinated the study, managed translation and cognitive debriefing activities for the USA and Canada, and helped draft the manuscript. DMM performed the Rasch analysis and managed the traditional statistical analysis. JT performed the traditional statistical analysis. DR assisted in the conceptualization and planning of the data analyses and with manuscript preparation and review. RWL and MPL contributed to the coordination of the analysis and the preparation of the manuscript. All authors reviewed the manuscript critically for content and approved it for submission.
